# Carnivorous plant evolution: is a killer defense always the best option?

**DOI:** 10.1093/jxb/erad431

**Published:** 2023-12-20

**Authors:** Carl Procko, Joanne Chory

**Affiliations:** Plant Biology Laboratory, Salk Institute for Biological Studies, 10010 N. Torrey Pines Rd, La Jolla, CA 92037, USA; Plant Biology Laboratory, Salk Institute for Biological Studies, 10010 N. Torrey Pines Rd, La Jolla, CA 92037, USA; Howard Hughes Medical Institute, Chevy Chase, MD, USA

**Keywords:** Carnivorous plants, evolution, jasmonates, prey digestion

## Abstract

This article comments on:

Pavlovič A, Koller J, Vrobel O, Chamrád I, Lenobel R, and Tarkowski P. 2024. Is the co-option of jasmonate signalling for botanical carnivory a universal trait for all carnivorous plants? Journal of Experimental Botany 75, 334–349.

**We don’t often think of plants as hunters. Yet, for a small but diverse group of flesh-eating plants, evolution has crafted them into skilled predators. Indeed, the leaves of plant carnivores have evolved snapping motions, hollow cage-like cavities, sticky secretions, and even suction power—all for the purpose of capturing small animals to provide nutrients not otherwise easily obtained from the nutrient-poor soils in which they grow. How these remarkable plants have evolved these killer abilities has long intrigued the scientific community and beyond. In this issue, Pavlovič *et al.* (2024) suggest that the evolutionary routes to carnivory may actually be broader than first thought.**

Botanical carnivory has evolved more than once, with at least 11 different origins spread across 20 genera in six plant orders (Fleischmann *et al.*, 2018; Lin *et al.*, 2021). This polyphyletic origin makes the plants ideally suited for studies of convergent evolution: in how many ways can you evolve a killer plant? Traditionally, these studies were limited to descriptions of leaf trap morphologies and their mechanisms of catching prey. However, for a plant to be carnivorous, it not only must catch animal prey—typically insects or other small arthropods—but must also digest the prey and acquire the nutrients. Recent technological advances in molecular biology have facilitated studies that now probe these other aspects of the carnivorous syndrome and, in particular, prey digestion.

Sometimes, digestion in plant carnivores is achieved by other organisms that interact with the plant, such as microbes or mutualistic arthropods (Fleischmann *et al.*, 2018). However, in many genera, digestion is achieved by the plant itself via the secretion of a mix of hydrolytic enzymes from specialized digestive glands (Schulze *et al.*, 2012; Fukushima *et al.*, 2017). These enzymes, which include chitinases, proteases, lipases, and phosphatases, are induced in non-carnivorous plants during defense responses; for example, to defend against fungal pathogens (Schlumbaum *et al.*, 1986; Zhao and Chye, 1999). What then regulates the expression of these pathogenesis-related proteins in plant carnivores, and has this molecular pathway been selected for carnivory independently across diverse plant groups? This is the question addressed by Pavlovič *et al*. (2024).

Molecular phylogenetics has dated the evolution of the oldest known lineage of plant carnivores to ~95 million years ago, within the order *Caryophyllales* (Fleischmann *et al.*, 2018). This lineage includes the Venus flytrap (*Dionaea muscipula*), with its bilobed, touch-sensitive snap-trap leaves, as well as sundews of the *Drosera* and pitcher plants of the *Nepenthes* genera, with adhesive and pitfall trap leaves, respectively. In these plants, the synthesis and secretion of digestive enzymes is regulated by prey capture (Bemm *et al.*, 2016; Pavlovič and Mithöfer, 2019). Extensive studies over the last two decades have shown that the molecular link between prey recognition and enzyme secretion is the jasmonate (JA) signaling pathway (Pavlovič and Mithöfer, 2019). JAs are small, lipid-based phytohormones best known for their role in defense against herbivores and pathogens in non-carnivorous plants. Studies using non-carnivorous *Arabidopsis thaliana* (Arabidopsis) have revealed that electrical and calcium signals following herbivore attack lead to an increase in JA biosynthesis, both at the wounded site and in distal leaves (Mousavi *et al.*, 2013; Toyota *et al.*, 2018). Ultimately, JA signaling alters gene expression, including the induction of chitinases and many other pathogenesis-related genes (Zhao and Chye, 1999). A similar story unfolds in carnivorous *Caryophyllales*: electrical and calcium signals generated by prey are correlated with increases in JA biosynthesis and JA-dependent gene expression (Escalante-Pérez *et al.*, 2011; Nakamura *et al.*, 2013; Procko *et al.*, 2022). Importantly, exogenous application of a JA analog can bypass prey feeding and recapitulate many aspects of the carnivorous syndrome, including digestive enzyme production (Escalante-Pérez *et al.*, 2011).

This co-option of the JA pathway towards the regulation of prey digestion in *Caryophyllales* has in part led to the hypothesis that botanical carnivory evolved from an insect and pathogen defense pathway (Pavlovič and Mithöfer, 2019). This is perhaps not so surprising; for example, secretory glands—including glandular hairs, or trichomes—are common in the non-carnivorous sister clade to carnivorous *Caryophyllales* and are very similar to digestive glands (Heubl *et al.*, 2006; Fleischmann *et al.*, 2018). Such glandular hairs are well known to provide chemical and structural defense to insect predation, synthesizing poisons and secreting sticky exudates to deter and impede small herbivores. Indeed, it has been hypothesized that the ancestral pre-adapted population that gave rise to modern-day *Caryophyllales* carnivores had these sticky, glandular hairs, which would facilitate both digestion and prey capture via adhesion (Heubl *et al.*, 2006). However, any link between glandular hair stimulation—whether mechanical or chemical—and JA biosynthesis has not been well explored outside of carnivorous plants. Despite this, in light of these morphological, physiological, and molecular similarities between plant carnivory and defense, we can forgive the 18th century poet-naturalist Erasmus Darwin for erroneously writing in his work *The Botanic Garden* that the sticky secretions from hairs of the sundew plant were not for attack but rather to protect the leaves from insect herbivores. It was his grandson, Charles Darwin, who supposed that glandular hairs could also be a first rung on the evolutionary ladder towards botanical carnivory (Darwin, 1875).

This hypothesis of repurposing the JA defense pathway towards carnivory is certainly an attractive one. However, while likely to be true for carnivorous *Caryophyllales*, is this a common route through which carnivory has evolved in other plant lineages? Pavlovič *et al.* now suggest that the answer to this question is an emphatic ‘no’.

To address this question, the authors looked at two different types of pitcher plants: the purple trumpet pitcher plant *Sarracenia purpurea*, from the order *Ericales*, and the independently evolved Australian pitcher plant *Cephalotus follicularis* (Cephalotus), of the order *Oxalidales*. Both species use ‘pitfall’ pitcher-shaped leaves for catching prey and exhibit digestive enzyme activity that bears a close resemblance to that seen in the carnivorous *Caryophyllales* (Fukushima *et al.*, 2017). Unlike the touch-sensitive Venus flytrap and sundews, Pavlovič and his colleagues found that purple and Cephalotus pitcher plants do not display obvious electrical signaling in response to prey capture. However, as the authors write, this in itself does not exclude the possibility of JA involvement for carnivory; for example, other genera within the Caryophyllales—specifically *Nepenthes* and *Drosophyllum*—also appear to lack robust and/or detectable electrical impulses in response to prey perception but do employ JAs for regulating digestive enzyme production (Yilamujiang *et al.*, 2016; Pavlovič *et al.*, 2024). In these plants, a requirement for electrical impulses may have been lost over time. More strikingly, the purple and Cephalotus pitcher plants fail to produce a detectable increase in JAs following prey capture. In contrast, mechanical wounding caused measureable changes in JA levels, similar to non-carnivorous plants.

While this work is understandably not an exhaustive look at all carnivorous plant genera, it is striking that to date the involvement of the JA pathway in regulating digestive enzymes in response to prey has only been observed in the *Caryophyllales* (Fig. 1). Is, therefore, digestive enzyme secretion in other plant carnivores no longer dependent on prey perception? While Pavlovič *et al*. suggest that this may be true for Cephalotus, it is likely that prey perception does regulate some enzymatic activity in the purple pitcher plant. Here, the pitcher is open to rainfall that may flush away valuable proteins inside, and which may thus necessitate controlled release of digestive enzymes only as needed. Furthermore, previous work from these same authors, and on which they build their current study (Kocáb *et al.*, 2020), showed that the carnivorous genus *Pinguicula* of the order *Lamiales* has strongly inducible secretion of pathogenesis-related enzymes which are also independent of JAs. Thus, it seems that these plants probably employ a signal transduction pathway linking prey perception to enzyme biosynthesis and secretion that is not JA based. But if not JAs, then what is this signaling intermediate? To date, the involvement of other phytohormones in carnivorous responses has not been well established, including for other defense-related hormones such as salicylic acid. Why (or how) these other phytohormones would independently regulate genes typically within the domain of JA defense responses is unclear. The answer then to this question remains unresolved.

**Fig. 1.**
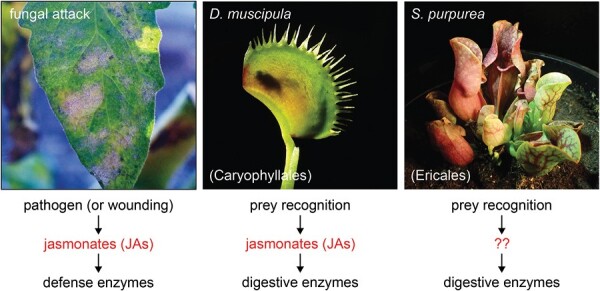
Co-option of defense-associated genes for prey digestion in carnivorous plants. Powdery mildew fungal infection of a non-carnivorous tomato plant leaf (*Solanum lycopersicum*; left), the snap-trap leaves of the Venus flytrap (*Dionaea muscipula*, order *Caryophyllales*; middle), and pitfall trap leaves of the purple pitcher plant (*Sarracenia purpurea*, order *Ericales*; right). Both carnivorous plant species have co-opted one aspect of plant defense during their independent evolution towards carnivory: the expression of pathogenesis-related hydrolytic enzymes. During defense in non-carnivorous plants, these enzymes help protect against fungal pathogens and insect herbivores, and are typically regulated by jasmonate (JA) hormones (left). In botanical carnivory, these enzymes function in digestion of animal prey. The expression of these enzymes is regulated by prey perception in both carnivorous species; however, only the Venus flytrap and other carnivores of the order *Caryophyllales* regulate enzyme expression by also co-opting the JA signaling pathway.

Not only does it seem that JAs are not required for the induction of digestive enzymes outside the *Caryophyllales*, but Pavlovič *et al*. also demonstrate that the expression of these genes in non-*Caryophyllales* pitcher plants is unaffected by exogenous treatment with a JA analog. This is despite the ancestral role of these genes in defense processes. Could there be a benefit to this decoupling of JA signaling and pathogenesis-related hydrolytic enzyme expression in plant carnivores? It is possible to imagine a scenario where carnivory could be in conflict with JA-mediated defenses, which must respond to and protect the plant from environmental stressors independently of whether a trap has caught an animal meal or not. In this scenario, it is beneficial for the plant to use different regulatory pathways: one for defense and the other for prey digestion. Likewise, in Arabidopsis plants, the electrical signals that induce JA biosynthesis in response to herbivory are systemic, initiating defense responses in leaves distal to the site of attack (Mousavi *et al.*, 2013). While systemic electrical and JA signaling probably confers an advantage when protecting oneself from herbivores or pathogens that can easily move between leaves, it does not benefit a plant carnivore, which requires only a local response at the site of prey capture. Indeed, it is noteworthy that electrical signals generated by prey in touch-sensitive Venus flytrap and sundews do not advance beyond the stimulated leaf (Williams and Pickard, 1972). It is conceivable that in these carnivorous *Caryophyllales*, the JA-regulated hydrolytic enzymes play a dual role, both in defense against pathogens and for prey digestion. However, in non-*Caryophyllales*, it would seem that the purpose of many is restricted to JA-independent carnivorous functions.

Undoubtedly, massively parallel sequencing technologies—which have revealed insights into carnivorous plant genomes and in particular trap transcriptomes and their regulation (Bemm *et al.*, 2016; Fukushima *et al.*, 2017)—as well as recent advances in carnivorous plant transformation and targeted genetic approaches (Procko *et al.*, 2023)—will continue to be instrumental in resolving these open questions. However, for now it seems that the field must rethink how it extrapolates lessons learned about botanical carnivory from the well-studied *Caryophyllales* to other carnivorous plant groups. Indeed, as Pavlovič *et al*. well conclude, it seems that co-option of JA defense signaling to carnivory in the *Caryophyllales* is perhaps the anomaly and not the norm; rather, there are more ways than one to evolve a killer plant.
